# Clinical and genetic profiling of nevoid basal cell carcinoma syndrome in Korean patients by whole-exome sequencing

**DOI:** 10.1038/s41598-020-80867-0

**Published:** 2021-01-13

**Authors:** Boram Kim, Man Jin Kim, Keunyoung Hur, Seong Jin Jo, Jung Min Ko, Sung Sup Park, Moon-Woo Seong, Je-Ho Mun

**Affiliations:** 1 Department of Laboratory Medicine, Seoul National University Hospital, Seoul National University College of Medicine, 101 Daehak-ro, Jongno-gu, Seoul, 03080 South Korea; 2 Department of Dermatology, Seoul National University Hospital, Seoul National University College of Medicine, 101 Daehak-ro, Jongno-gu, Seoul, 03080 South Korea; 3 Institute of Human-Environment Interface Biology, Seoul National University, Seoul, 03080 South Korea; 4 Department of Pediatrics, Seoul National University Children’s Hospital, Seoul National University College of Medicine, Seoul, 03080 South Korea; 5 Cancer Research Institute, Seoul National University College of Medicine, Seoul, 03080 South Korea

**Keywords:** Skin cancer, Next-generation sequencing

## Abstract

Nevoid basal cell carcinoma syndrome (NBCCS) is mainly characterised by multiple basal cell carcinomas (BCCs) caused by *PTCH1, PTCH2,* and *SUFU*. However, clinical and genetic data on Asian NBCCS patients are limited. We aimed to analyse the clinical phenotypes and genetic spectrum of Korean patients with NBCCS. Fifteen patients with NBCCS at Seoul National University Hospital were included, and their clinical data were analysed. Whole-exome sequencing and/or multiplex ligation-dependent probe amplification using peripheral blood were performed to identify genetic causes. Genetic analysis revealed that 73.3% (11/15) of the patients carried 9 pathogenic variants, only in the *PTCH1* gene. Variants of uncertain significance (VUS) and likely benign were also detected in 2 (13.3%) and 2 (13.3%) patients, respectively. BCCs were found in the majority of the cases (93.3%) and the number of BCCs increased with age (ρ = 0.595, P = 0.019). This study revealed that *PTCH1* pathogenic variants were the main cause of NBCCS in Korean patients. As BCCs are commonly detected, a periodic dermatologic examination is recommended. Finally, our results support the addition of genetic screening to the existing criteria for NBCCS diagnosis.

## Introduction

Nevoid basal cell carcinoma syndrome (NBCCS, MIM 109400), also known as Gorlin syndrome, is a rare autosomal dominant disorder predisposing to multiple basal cell carcinomas (BCCs)^1^. It is characterised by BCCs, odontogenic keratocysts, palmoplantar pits, falx cerebri calcification, and other developmental anomalies^2^. The diagnostic criteria for NBCCS proposed by Kimonis et al. in 1997 are still widely used^3^. When either two major, or one major and two minor criteria are fulfiled, the diagnosis of NBCCS can be confirmed.

NBCCS is mainly caused by variants in *PTCH1*—the human homolog of the *Drosophila patched* gene^4^. It is a tumour suppressor gene of the Sonic hedgehog (SHH) signalling pathway which encodes the transmembrane glycoprotein, patched-1. Loss-of-function variants of *PTCH1* cause abnormal constitutive upregulation of the pathway and development of BCC. Additionally, *PTCH2* and *SUFU* variants are known to cause NBCCS in rare cases^5^. The detection rate of *PTCH1* variants in clinically diagnosed NBCCS was reported to range from 40 to 85%, while *SUFU* variants were found in 5.3% of all NBCCS patients^6^. However, the genomic characteristics of NBCCS in Asian descents remain largely unknown.

Although the prevalence of NBCCS is approximately 1 in 56,000–256,000, the incidence of the syndrome in Asia is uncertain^7,8^. In previous studies, the prevalence of NBCCS was reported to be 1 in 235,800 in Japan and 1 in 13,939,393 in Korea^9,10^. A systematic review of NBCCS literature revealed that significant differences exist between ethnic groups. In Northern Europe, patients showed significantly higher frequencies of BCCs, falx cerebri calcification, palmar and plantar pits, and family history. East Asians displayed significantly higher frequencies of keratocystic odontogenic tumours, cleft lips and palates, and hypertelorism^11^. A previous study describing a pooled analysis of Korean patients with NBCCS reported a high frequency of odontogenic keratocysts (90.9%), but a low BCC detection rate (15.2%)^10^.

In this study, we aimed to analyse clinical phenotypes and characterise the genetic profiles of Korean patients with NBCCS by whole-exome sequencing (WES) and multiplex ligation-dependent probe amplification (MLPA).

## Results

### Clinical characteristics of patients with NBCCS

A total of 15 patients were included. The age distribution of patients with NBCCS was 9–66 years (median 34 years) and the age of onset was 8–51 years (median 19 years). The male to female ratio was 1:2.75, although NBCCS is known to present equally in both males and females^12^.

Unlike previous studies which showed that BCCs accounted for 15.2–37.8% of NBCCS cases in Asian patients, our data demonstrated that most patients (93.3%) had at least one BCC^10,13,14^. Specifically, more than 2 BCCs were found in 86.7% of patients, while BCC onset before the age of 20 was observed in 46.7% of patients. The number of BCCs increased with age (ρ = 0.595, P = 0.019). In our cohort, BCCs were generally managed successfully with surgical excision. For small BCCs, punch biopsies were used to minimise normal skin-loss. CO_2_ laser ablation was used for multiple superficial BCCs. BCC went undetected in 1 patient only (case 14). She was diagnosed with NBCCS with multiple odontogenic cysts and palmoplantar pits at 12 years old. Follow-up examinations did not reveal any evidence of BCC until the age of 19 years.

**Figure 1:**
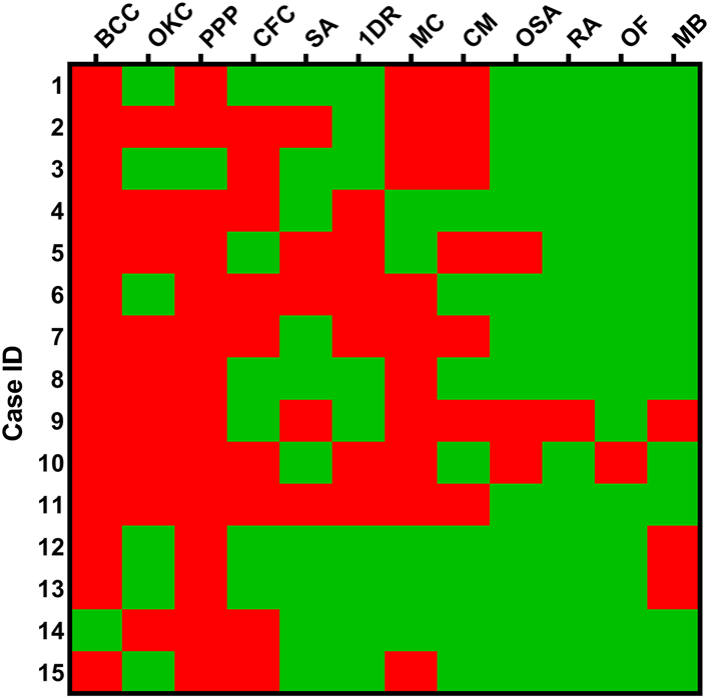
Heatmap of 15 NBCCS cases versus clinical features according to the criteria set by Kimonis et al.^3^. Clinical features are indicated in red (present) and green (absent). *NBCCS* nevoid basal cell carcinoma syndrome, *BCC* more than two basal cell carcinomas or one under the age of 20 years, *OKC* odontogenic keratocysts, *PPP* three or more palmar or plantar pits, *CFC* lamellar calcification of the falx cerebri, *SA* bifid, fused, or markedly splayed ribs, *1DR* first-degree relative with nevoid basal cell carcinoma syndrome, *MC* macrocephaly, *CM* congenital malformations, *OSA* other skeletal abnormalities, *RA* radiological abnormalities, *OF* ovarian fibroma, *MB* medulloblastoma.

Palmar or plantar fits were found in majority of the cases (93.3%), followed by macrocephaly (66.7%), odontogenic keratocysts (60.0%), lamellar calcification of the falx cerebri (60.0%), congenital malformations (46.7%), first degree relative with NBCCS (40.0%), bifid, fused or markedly splayed ribs (33.3%), ovarian fibroma (6.7%) and radiologic abnormalities (6.7%; Table 1 and Fig. 1). Medulloblastoma was detected in 3 patients (20.0%). Case 3 had a meningioma, which was not included in the diagnostic criteria but often presents in NBCCS^15^.

**Table 1:** Diagnostic criteria for NBCCS and clinical phenotype of 15 Korean patients.

Diagnostic criteria for NBCCS	N (%)
**Major criteria**	
1. More than two BCCs or one under the age of 20 years	14 (93.3)
2. Odontogenic keratocysts of the jaw	9 (60.0)
3. Three or more palmar or plantar pits	14 (93.3)
4. Lamellar calcification of the falx cerebri	9 (60.0)
5. Bifid, fused or markedly splayed ribs	5 (33.3)
6. First degree relative with NBCCS	6 (40.0)
**Minor criteria**	
1. Macrocephaly determined after adjustment for height	10 (66.7)
2. Congenital malformations	7 (46.7)
3. Other skeletal abnormalities	3 (20.0)
4. Radiological abnormalities	1 (6.7)
5. Ovarian fibroma	1 (6.7)
6. Medulloblastoma	3 (20.0)
Total	15 (100.0)

*NBCCS* nevoid basal cell carcinoma syndrome, *BCC* basal cell carcinoma.

All patients fulfiled the criteria proposed by Kimonis et al.^3^ during clinical examination, except for one child (case 1). This patient fulfiled one major and one minor criterion (palmoplantar pits and macrocephaly) at the first visit (age 8 years), which was insufficient to meet the diagnostic criteria. Due to clinical suspicion, genetic analysis was performed. The results revealed whole gene deletion of *PTCH1*. Therefore, we performed detailed physical and dermatologic examinations using dermoscopy. The patient displayed multiple, 1–2 mm-sized brown papules on the abdomen. Dermoscopic examination revealed patterns associated with BCC. A subsequent punch biopsy confirmed the presence of BCCs. In addition, hypertelorism was detected by measuring the interpupillary distance.

### Genetic profiles of patients with NBCCS

**Figure 2:**
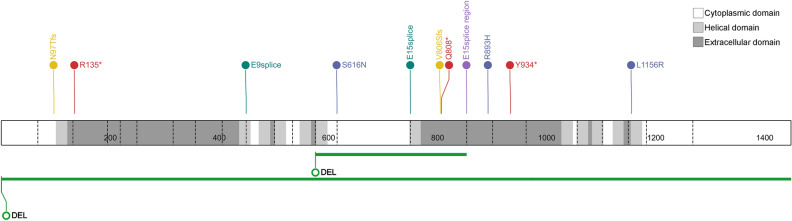
*PTCH1* variant distribution of 15 Korean patients with NBCCS along with the patched-1 protein. *DEL* deletion, *red* nonsense variant, *yellow* frameshift variant, *green* large deletion, *dark green* splice site variant, *blue* missense variant, *purple* splice region variant.

Of the 15 NBCCS patients, we found 4 nonsense, 4 splice site, 3 missense variant, 2 frameshift, 1 partial deletion, and 1 gross deletion. When we categorized them according to the American College of Medical Genetics and the Association for Molecular Pathology (ACMG/AMP) guidelines^16^, 9 *PTCH1* pathogenic variants (including likely pathogenic variants) were found in 11 patients (73.3%). Variants of uncertain significance (VUS) and likely benign were also detected in 2 (13.3%) and 2 (13.3%) patients, respectively (Table 2). All variants were located along *PTCH1* and scattered without mutational hotspot (Fig. 2). However, none of the patients had *PTCH2* or *SUFU* pathogenic variants.

**Table 2:** *PTCH1* variants of 15 NBCCS patients.

Case	Sex/age	DNA variants	Protein alteration	Location	Variant type	Classification	ACMG criteria	References
1	F/9	del 9q22.31–q22.33		Whole gene	Gross deletion	Pathogenic		Matsudate et al.^17^
2	M/37	Exon 13–15 deletion		Exon 13–15	Partial deletion	Pathogenic		Novel
3	M/56	c.290delA	p.Asn97Thrfs*20	Exon 2	Frameshift	Pathogenic	PVS1, PM2, PP5	Wilson et al.^18^
4	F/62	c.403C > T	p.Arg135*	Exon 3	Nonsense	Pathogenic	PVS1, PM2, PP5	Wicking et al.^19^
5	F/22	c.403C > T	p.Arg135*	Exon 3	Nonsense	Pathogenic	PVS1, PM2, PP5	Wicking et al.^19^
6	F/66	c.1347 + 1G > A	p.?	Intron 9	Splice site	Pathogenic	PVS1, PM2, PP5	Reinders et al.^20^
7	F/38	c.1347 + 1G > A	p.?	Intron 9	Splice site	Pathogenic	PVS1, PM2, PP5	Reinders et al.^20^
8	F/24	c.1847G > A	p.Ser616Asn	Exon 13	Missense	VUS	PM2, PP3	Novel
9	M/13	c.2251-3C > G	p.?	Intron 14	Splice site	VUS	PM2, PP3, BS3	Sun JS et al.^21^
10	F/35	c.2415dup	p.Val806Serfs*23	Exon 15	Frameshift	Likely pathogenic	PVS1, PM2	Novel
11	F/20	c.2422C > T	p.Gln808*	Exon 15	Nonsense	Likely pathogenic	PVS1, PM2	Waszak et al.^22^
12	F/34	c.2560 + 7C > T	p.?	Intron 15	Splice region	Likely benign	BS1, BP6	rs75576651
13	F/19	c.2678G > A	p.Arg893His	Exon 16	Missense	Likely benign	BS1, BP6	Tate et al.^23^
14	F/19	c.2802T > A	p.Tyr934*	Exon 17	Nonsense	Likely pathogenic	PVS1, PM2	Novel
15	M/60	c.3467T > G	p.Leu1156Arg	Exon 21	Missense	Likely pathogenic	PM2, PM6, PP3, PP5	Gianferante et al.^24^

*VUS* variant of uncertain significance, *PVS* pathogenic very strong, *PM* pathogenic moderate, *PP* pathogenic supporting, *BS* benign strong, *BP* benign supporting.

Of the 15 patients, 6 (40.0%) had a family history of NBCCS and their variants were confirmed as familial variants with pedigree analysis. Cases 4, 5 and 6, 7 had a mother–daughter relationship and the same pathogenic variants. There were 3 nonsense, 2 frameshift, 1 splice site and 1 missense variant among the pathogenic sequence variants, which is in line with a recent review article demonstrating the most common variant form is frameshift, followed by nonsense variant^25^. Among these, two pathogenic variants (c.2415dup and c.2802T > A) were considered novel, and one variant (c.2422C > T) was not reported in NBCCS, but in medulloblastoma^22^. Although copy number alterations have been reported as rare, 2 patients (13.3%) in our cohort showed gross or partial deletion of *PTCH1*^24^.

Two variants (c.1847G > A and c.2251-3C > T) were classified as VUS (Table 2). Both were not observed in the normal population (PM2) and in silico tools predicted them to be pathogenic (PP3). However, Sun et al. previously showed that c.2251-3C > T had no splicing effect, using RT-PCR (BS3)^21^. As no further information on the other variant (c.1847G > A) was available, we classified it as VUS.

## Discussion

In this study, we described the clinical phenotypes and genetic profiles of 15 Korean patients with NBCCS. While the genetic background of NBCCS was first revealed in 1992, the genetic spectrum in Asian patients remains unknown^26^. Because *PTCH1* has 24 exons, WES can be used for variant analysis, making it possible to analyse additional genes, like *PTCH2* and *SUFU*. Therefore, we used WES for the genetic analysis of NBCCS. Additionally, we utilised MLPA for the detection of *PTCH1* copy number variants.

Previous studies have shown that the frequency of BCC ranges from 15.2 to 51.4% in Asians^9,10,13^. However, we detected BCC in all except one NBCCS case (93.3%). The difference between our data and those of previous studies is that this study was conducted in clinics by experienced dermatosurgeons with expertise in skin cancers. Previous studies were conducted in dental clinics^10,27^. Selection and observation bias may therefore exist in previous literature.

We found 9 patients had *PTCH1* pathogenic single nucleotide variants and 2 patients had large deletions, so exonal deletions of *PTCH1* accounted for 13.3% of our cohort. According to previous Japanese data, 3 out of 20 patients (13.6%) were reported to have *PTCH1* gross deletions with chromosomal microarray analysis^17^. Also, large genomic deletions and duplications were found in 8% of the 110 new variants in European cohorts^20^. Therefore, copy number analysis of *PTCH1* as well as sequence analysis are necessary for the genetic consultation of patients with NBCCS.

Since NBCCS was first described by Gorlin and Goltz in 1960, several groups have recommended guidelines for the diagnosis of NBCCS^2^. Among these, the criteria proposed by Kimonis et al.^3^ are most widely used. Based on these criteria, we successfully diagnosed patients with NBCCS, except for one young patient (case 1) who initially did not fulfil the criteria. Based on clinical suspicion of the syndrome, a molecular test was performed and *PTCH1* deletion was detected. According to the consensus statement from the first international colloquium on NBCCS, genetic confirmation with one major criterion can be used to make NBCCS diagnosis^28^. Because some features tend to occur in older NBCCS patients, this criterion would be particularly helpful for young patients, as in our case^29^. Since early detection and timely management of BCCs can result in the prevention of deformities with minimal scarring, genetic testing might be essential in younger patients.

Genotype–phenotype correlations in NBCCS have not been evident in previous studies^19,30,31^. In a previous study, the risk for medulloblastoma was higher in *SUFU*-related NBCCS cases than in *PTCH1*-related NBCCS patients^6^. Specifically, medulloblastoma was found in 3 out of 9 individuals (33.3%) with *SUFU* variants, but in only 2 out of 115 patients (1.7%) with *PTCH1* variants. In our study, 20% of patients had medulloblastoma, but no *PTCH1* pathogenic variants were detected. Our results, therefore, supported that *PTCH1*-related NBCCS patients have a relatively low risk for medulloblastoma.

The management of NBCCS should be tailored according to each patient’s condition. Various specialists should participate in treating the patients, including dermatologists, oral or dental surgeons, paediatricians, plastic surgeons and medical geneticists. As BCC has a prevalence of 47–96%, early detection and treatment is essential to minimise cosmetic disfigurement^32^. Although surgeries like wide excision or Mohs micrographic surgery are used for high-risk BCCs or destructing/disfiguring lesions, they are often not suitable for patients with NBCCS with multiple and extensive lesions as they can be time-consuming and deforming^1^. In patients in our study, BCCs were generally well managed with surgery, except for one case (case 6). This patient displayed multifocal recurrence where she received a full-thickness skin graft after BCC removal. Hence, we suggest that skin grafting be avoided as it makes it difficult to detect recurrence in patients with NBCCS.

A limitation of our study is the small sample size because of the low prevalence of NBCCS in Korea. Only patients of Korean descent were included. Patients presenting with similar and different features from the other population need to be investigated and a larger prospective multinational cohort study should be conducted. Besides, the correlation of molecular profiles between cancer tissues and germline variants requires further investigation. Lastly, the overall detection rate of pathologic variants was 73.3%. In our study, we did not analyse copy number variation of other genes except *PTCH1*. In addition, there is a possibility of involvement of unknown candidate genes that account for Gorlin syndrome. Further studies about the SHH pathway-related genes and copy number analysis might be helpful for understanding the pathogenesis of those patients^33^.

In summary, we explored the clinical and genetic profiles of Korean patients with NBCCS and demonstrated the diagnostic utility of genetic testing by WES and MLPA. Additionally, we detected a high rate of BCC in Korean patients with NBCCS, as opposed to previous knowledge. This suggests the necessity of regular screening for BCC in Korean patients with NBCCS. Finally, our results underline the need for genetic diagnosis, particularly in younger patients. We, therefore, suggest the addition of genetic criterion to the existing diagnostic criteria for NBCCS.

## Methods

### Study population

Fifteen patients who fulfiled the diagnostic criteria for NBCCS set by Kimonis et al.^3^ were recruited for this study, which was conducted from 2017 to 2019 at Seoul National University Hospital. Among them, 4 patients from 2 families (cases 4, 5, and 6, 7) were included. Clinical information, including radiological examination and family history was collected from the hospital medical records. Written informed consent was obtained from all patients. This study was performed in accordance with the Declaration of Helsinki and was approved by the Institutional Review Board of Seoul National University Hospital (No. 1910-170-1074). This study was supported by the National Supporting Program for Genetic Diagnosis of Rare Diseases of the Korea Centers for Disease Control & Prevention.

### Whole-exome sequencing

Peripheral blood from patients was collected and stored in EDTA bottles and genomic DNA was extracted from each sample using the Gentra Puregene Blood kit (Qiagen, Hilden, Germany), according to the manufacturer’s protocol. The DNA concentration and purity were quantified using a NanoDrop spectrophotometer (Thermo Fisher Scientific, Waltham, MA, USA). DNA was sonicated using Covaris (Covaris, Inc., Woburn, MA, USA). Target enrichment was performed on the extracted DNA with SureSelect Human All Exon (version 5; Agilent, Santa Clara, CA, USA), for exome capture. Paired-end sequencing of all captured libraries was performed with the Illumina HiSeq 2000 or 2500 platform (Illumina, San Diego, CA, USA), as previously described^34^.

### Variant analysis

Bioinformatics analyses were performed by aligning to the human reference sequence, GRCh37/hg19, using a lab-developed pipeline based on NextGENe software version 2.4.0.1 (SoftGenetics, State College, PA, USA; https://softgenetics.com). The reference sequences of *PTCH1* (NM_000264.4), *PTCH2* (NM_003738.4), and *SUFU* (NM_016169.3) were used. The cut-off of variant allele frequency was 20% and the minimum read count was 10×. The Genome Aggregation Database and Korean Reference Genome Database were used to filter out benign variants. The Human Gene Mutation Database and ClinVar database were screened for previously reported variants. For in silico prediction, SIFT, Mutation Taster and PolyPhen2 were used as described previously^35^. Variants were classified as benign, likely benign, VUS, likely pathogenic and pathogenic, according to the ACMG/AMP guidelines^16^. The distribution of variants was analysed using ProteinPaint^36^.

### Deletion/duplication analysis

Gene dosage analysis of *PTCH1* was performed using samples that showed no pathogenic variants by WES analysis. MLPA was performed using SALSA MLPA Probemix P067-B3 (MRC-Holland, Amsterdam, The Netherlands), according to the manufacturer’s guidelines. In brief, 100 ng of genomic DNA was denatured for 5 min at 98 °C and hybridised with probes for 16 h at 60 °C. Ligation was done by ligase for 15 min at 54 °C and polymerase chain reaction of the ligated probes was done for 35 cycles using the Veriti 96-Well Thermal Cycler (Thermo Fisher Scientific). Each reaction was quantified using capillary electrophoresis with an ABI PRISM 3130xl Genetic Analyzer (Thermo Fisher Scientific). The results were analysed with the Coffalyser.Net version 140721.1958 (MRC-Holland; https://www.mrcholland.com/technology/software/coffalyser-net). We defined the normal range of the gene as 0.7–1.3, and deletion was identified when normalised peak ratio value was < 0.7 and duplication when > 1.3.

### Statistical analysis

Spearman’s correlation coefficient was used for correlation analysis, and data were analysed with SPSS version 25.0 (IBM, Armonk, NY, USA). All statistical analyses were two-tailed and P values < 0.05 were considered statistically significant.
